# Measles Outbreak in Macedonia: Epidemiological, Clinical and Laboratory Findings and Identification of Susceptible Cohorts

**DOI:** 10.1371/journal.pone.0074754

**Published:** 2013-09-10

**Authors:** Irena T. Kondova, Zvonko Milenkovic, Sanja P. Marinkovic, Golubinka Bosevska, Gordana Kuzmanovska, Goran Kondov, Sonja Alabakovska, Claude P. Muller, Judith M. Hübschen

**Affiliations:** 1 University Clinic of Infectious Diseases and Febrile Conditions, Clinical Centre, Medical Faculty, Skopje, R. Macedonia; 2 Institute for Public Health, Skopje, R. Macedonia; 3 University Clinic for Thoracovascular Surgery, Clinical Centre, Medical Faculty, Skopje, R. Macedonia; 4 Institute for Biochemistry, Medical Faculty, Skopje, R. Macedonia; 5 Institute of Immunology, Centre de Recherche Public de la Santé / Laboratoire National de Santé, Luxembourg, Luxembourg; University of Florida, United States of America

## Abstract

**Objectives:**

Despite a 92-99% national vaccination coverage since 2000, the former Yugoslav Republic of Macedonia experienced a large measles outbreak between 2010 and 2011. Here we investigate the characteristics of patients hospitalized during this outbreak at the Clinic of Infectious Diseases in Skopje.

**Methods:**

Epidemiological, clinical and laboratory data of 284 measles patients, including 251 from Skopje (43.80% of the 573 reported cases) and 33 from elsewhere in Macedonia were collected.

**Results:**

The most affected age groups were children up to 4 years of age and adolescents/adults of 15 years and older. Most patients were unvaccinated (n=263, 92.61%) and many had non-Macedonian nationalities (n=156, 54.93%) or belonged to the Roma ethnicity (n=73, 25.70%). Bronchopneumonia and diarrhea were the most common complications. Eighty-two out of 86 tested patients (95.35%) had measles-specific IgM antibodies. The outbreak was caused by the measles variant D4-Hamburg.

**Conclusions:**

The epidemic identified pockets of susceptibles in Skopje and indicated that additional vaccination opportunities in particular for people with non-Macedonian nationality and traveler communities are warranted to ensure efficient measles control in Macedonia. The high attack rate among children of less than 1 year suggests that vaccination before 12 months of age should be considered in high risk settings.

## Introduction

Before immunisation was possible, measles was a typical childhood disease and more than 90% of the population were immune by the age of 15 years. The disease can be associated with severe and sometimes fatal complications [1]. Despite a worldwide drop of 71% of measles-related deaths between 2000 and 2011, the virus continues to be responsible for about 158 000 deaths annually [2].

The World Health Organization European Region aims to eliminate measles and rubella by 2015 [3]. Despite considerable progress in measles control, outbreaks were reported from numerous European countries in 2010 and 2011 [4–10].

In the former Yugoslav Republic of Macedonia, the combined measles–mumps–rubella vaccine (MMR) is given at 12 months and again before school entry at 6 years of age. Coverage with both doses has been above 90% since 2000 (range 92-99%) [11] and only 1 to 36 cases were reported annually between 2000 and 2009 [12].

Between September 2010 and July 2011 the country experienced an unusually large measles outbreak in Skopje and other cities with many hospitalized patients. Here we describe epidemiological, clinical and laboratory characteristics of the measles patients hospitalized at the main infectious disease hospital of Macedonia during the 2010/2011 outbreak.

## Materials and Methods

**Figure 1:**
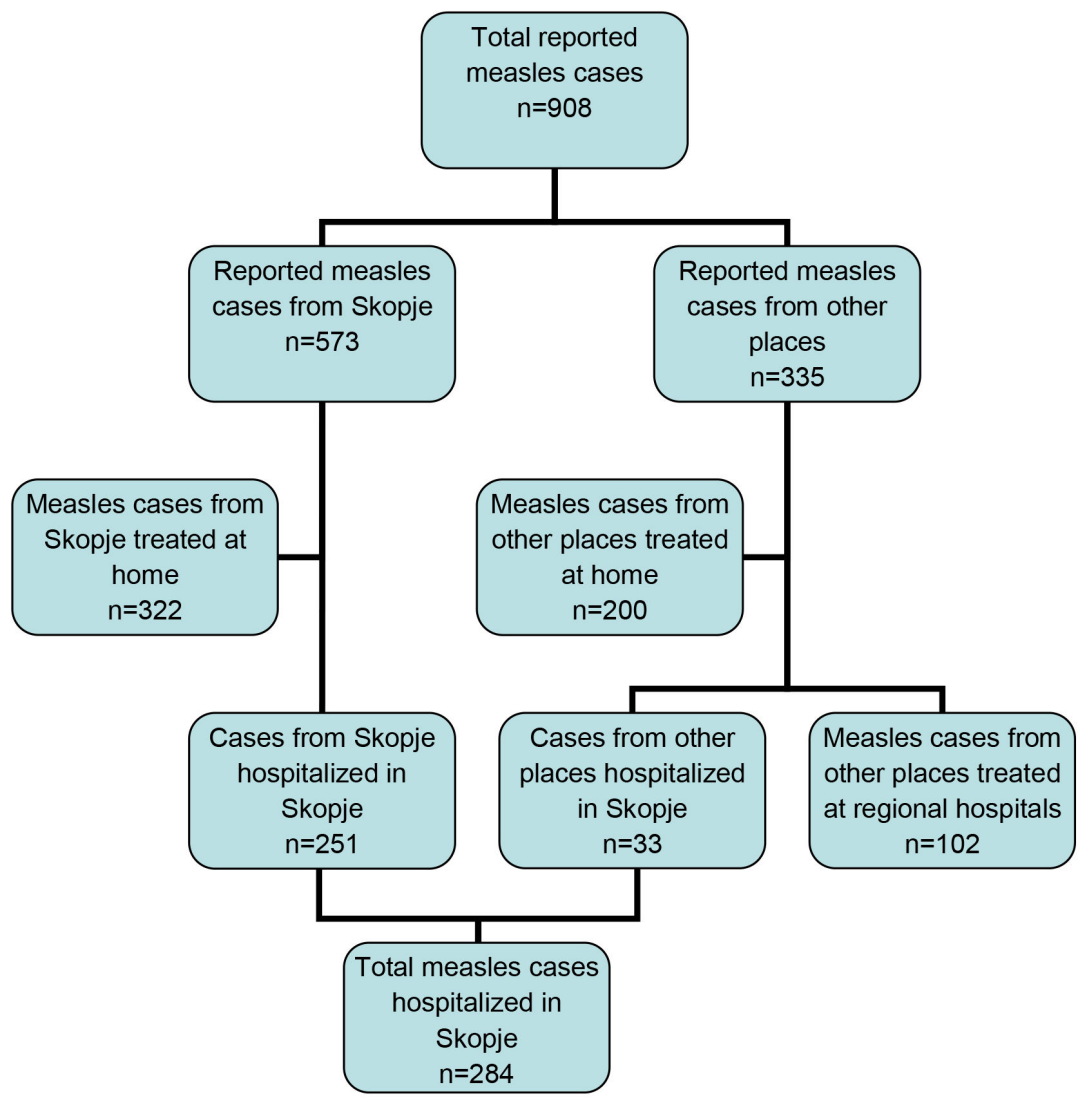
Diagram showing the different cohorts of measles patients reported during an outbreak in Macedonia, 2010-2011.

A total of 284 measles patients were hospitalized at the Clinic of Infectious Diseases and Febrile Conditions in Skopje between September 2010 and July 2011 (Figure 1). Clinical case definition included fever, a generalized maculopapular rash for at least three days and either cough, coryza or conjunctivitis [13]. Whenever cases occurred in the same city and within less than one maximum incubation period (18 days) apart, they were considered to be epidemiologically linked and related to the outbreak [14]. Laboratory confirmation was obtained by detection of measles-specific IgM antibodies in serum (n=86) using a commercial ELISA kit (Enzygnost®, Siemens). Serum, urine, and throat swabs from 18 patients were sent to the WHO European Regional Reference Laboratory in Luxembourg for confirmation of the outbreak and genotyping of the virus strains.

Statistical analyses were done with Microsoft Excel 2007 and Statistic 7.

**Table 1:** Characteristics related to hospitalization of the 284 measles cases.

**Characteristics related to hospitalization**	
	***Mean+ Standard Deviation***
Symptom duration before hospitalization	4.55 (±2.14 days)
Duration of antibiotic therapy at home	3.59 (±1.74 days)
Duration of antibiotic therapy in hospital	8.11 (±2.78 days)
Duration of fever	6.65 (±1.95 days)
Duration of hospitalization	8.68 (±2.75 days)
	***Number(%***)
Antibiotic therapy at home	151 (53.2%)
Antibiotic therapy during hospitalization	275 (96.8%)

### Ethics statement

Data obtained from the medical records of the patients were anonymized and analyzed retrospectively. All diagnostic methods and patient treatment were done according to the standard protocols for diagnosis and therapy of measles during epidemics. These protocols were established and approved by the institutional board of the University Clinic of Infectious Diseases and Febrile Conditions, Skopje, Republic of Macedonia and the Ministry of Health of the Republic of Macedônia.

## Results

**Figure 2:**
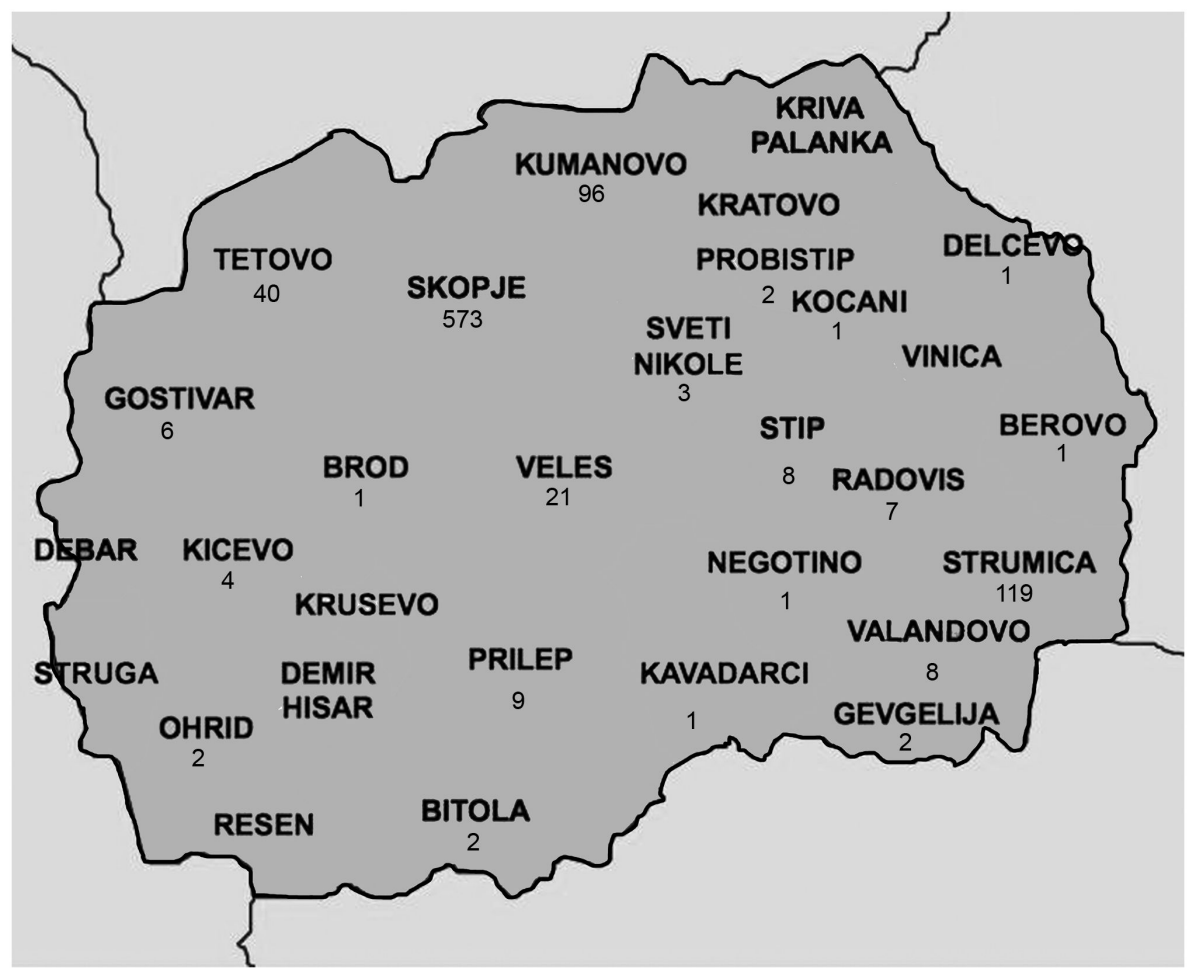
Number of measles cases in the different cities of Macedonia during an outbreak, 2010-2011.

In August 2010, the number of measles cases notified in Macedonia started to increase and subsequently outbreaks were reported from a number of cities. The first case occurred in Kumanovo on August 23, 2010. Until August 19, 2011, a total of 908 cases were reported from throughout the country. Most patients were from Skopje (n=573, 63.11%), followed by Strumica (n=119, 13.11%), Kumanovo (n=96, 10.57%), Tetovo (n=40, 4.41%) and Veles (n=21, 2.31%). The remaining patients (n=59, 6.50%) were from other towns or villages in Macedonia (Figure 2).

**Figure 3:**
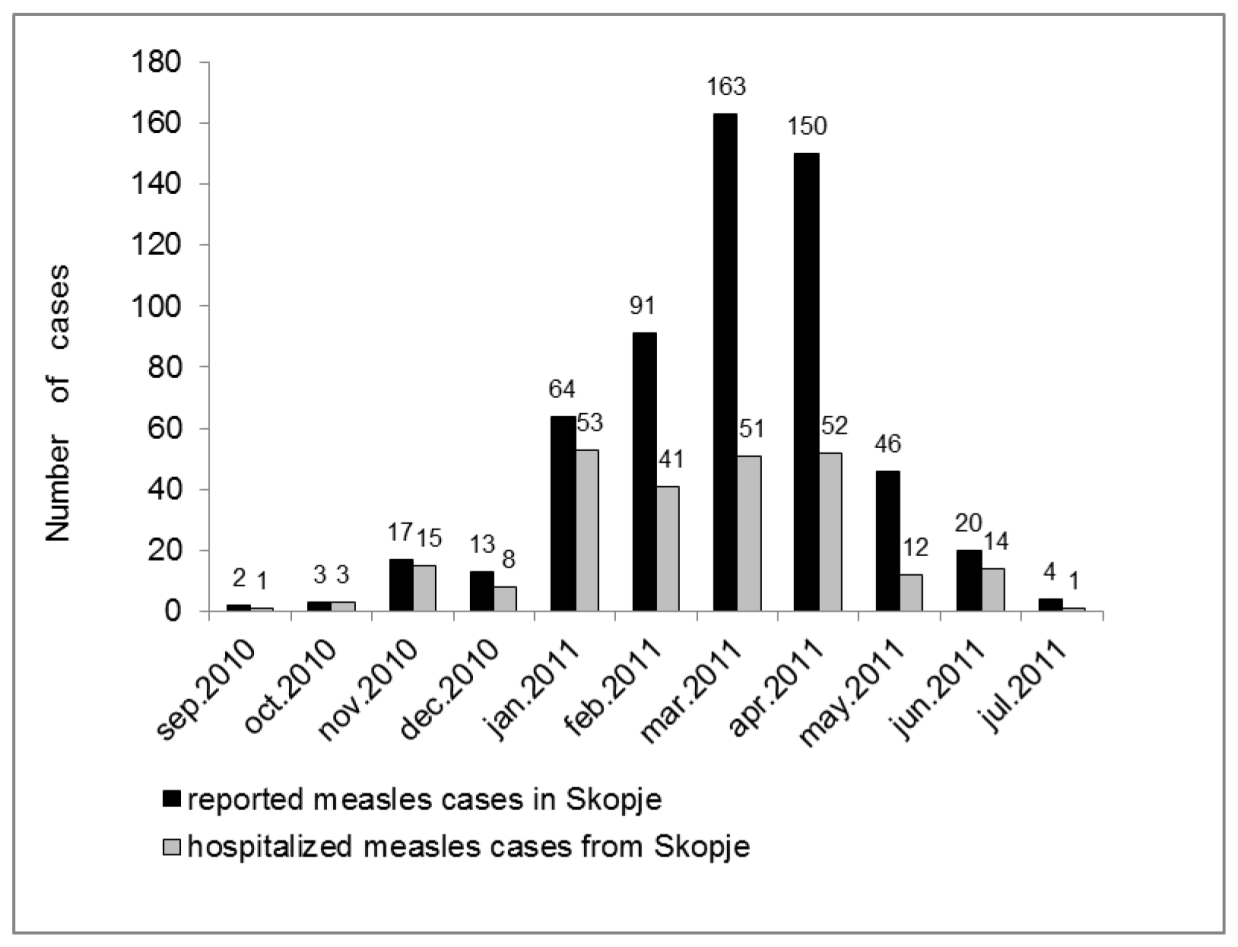
Reported and hospitalized measles patients from Skopje according to month between September 2010 and July 2011.

Between September 2010 and July 2011, a total of 284 measles patients were hospitalized at the Clinic of Infectious Diseases and Febrile Conditions in Skopje, with 251 of them being from Skopje (43.80% of the 573 reported cases) and 33 from other towns in Macedonia. Most cases from Skopje (n=197, 78.49%) were hospitalized between January and April 2011 (Figure 3).

**Figure 4:**
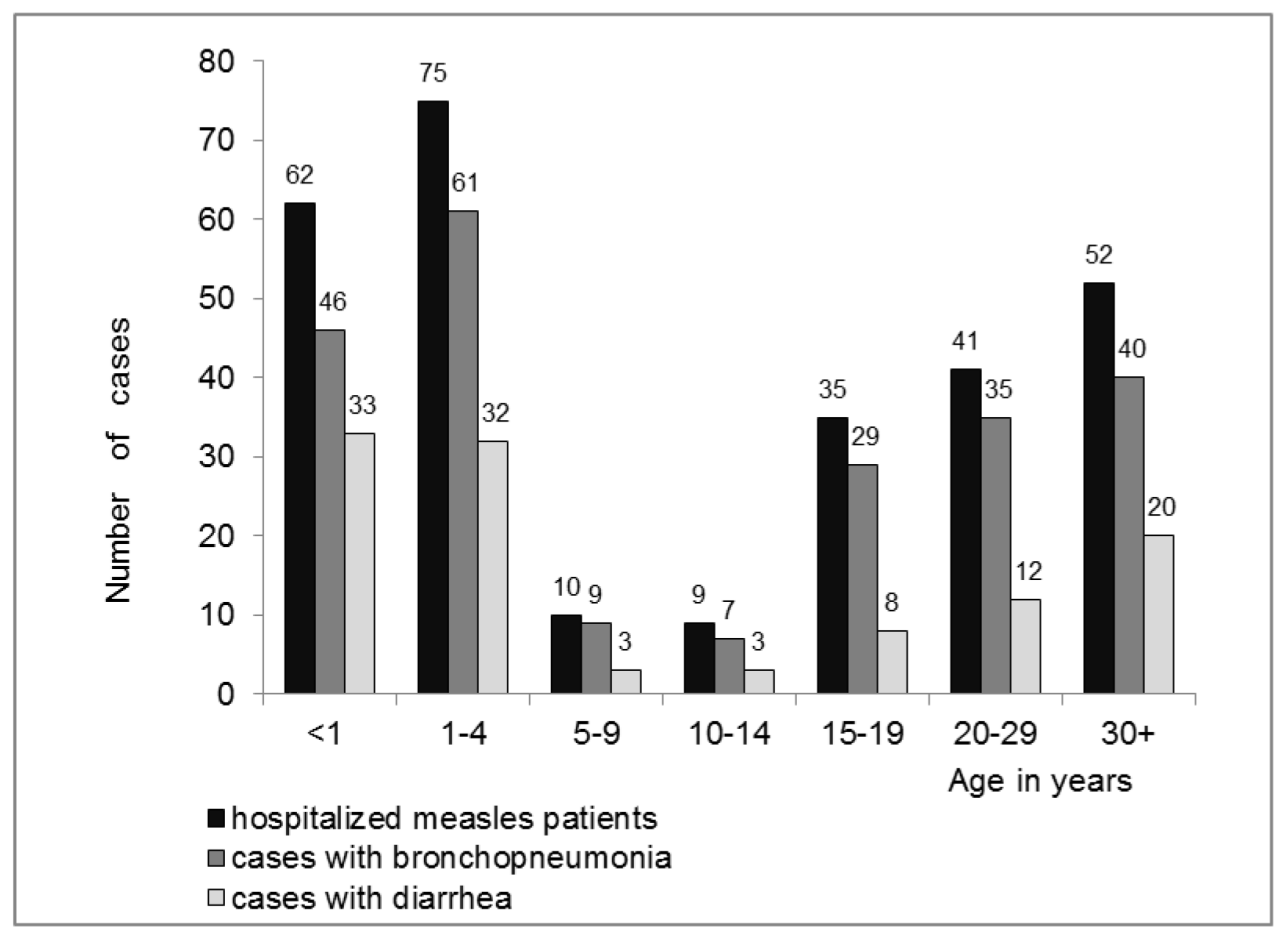
Measles patients hospitalized at the Clinic in Skopje, cases with bronchopneumonia and with diarrhea according to age-group.

Among the 284 patients, 144 (50.7%) were male and 140 (49.3%) female. The mean age was 13.2 years, with a range from <1 year up to 47 years. The most affected age groups were children up to 4 years of age and adolescents/adults of 15 years and older (Figure 4).

According to their vaccination cards, only 21 patients (7.39%) had previously been vaccinated. Nine of these patients were younger than 5 years and had thus received only a single dose. The other 12 patients were between 15 and 35 years old and except for one, they all had received only a single dose of measles containing vaccine.

Nearly half of the hospitalized patients were of Albanian nationality (n=137, 48.24%) and slightly less were Macedonian (n=128, 45.07%, including 73 Roma). The remaining patients (n=19, 6.69%) had various other nationalities such as Serb or Turk.

Of the 86 serum samples available for testing, 82 (95.35%) were positive for measles-specific IgM antibodies. Genotype information was obtained from 3 patients. The measles virus variant „D4-Hamburg“ (MVs/Hamburg. DEU/03.09, GenBank accession number HQ436108) was detected in all three. In addition to the 82 laboratory confirmed cases, 175 epidemiologically linked cases were hospitalized. The mean duration of clinical symptoms before hospitalization was about four and a half days and the mean duration of hospitalization was more than eight days (Table 1). 151 of the hospitalized patients (53.17%) took already antibiotics upon admission. During hospitalization 275 patients (96.83%) were treated with antibiotics. At the first medical consultation, all patients presented with symptoms consistent with the measles case definition. Most patients (n=227, 79.93%) had bronchopneumonia and nearly 40% (n=111, 39.08%) had diarrhea (Table 2 and Figure 4). Cough with expectoration was present in 32 patients (11.27%), most of them were adults of 20 years and older (25/32, 78.13%). Less common presentations included otitis (n=8, 2.82%; 7/8 patients or 87.50% were ≤ 4 years), laryngitis (n=8, 2.82%; 5/8 patients or 62.50% were ≥ 20 years), febrile convulsions (n=4, 1.41%; all of them among 1-4 year old children) and epistaxis (n=2, 0.70%, both patients were between 20 and 29 years old) (Table 2). Several patients had underlying health conditions such as chronic bronchitis (n=5), asthma (n=4), epilepsy (n=2) and psychomotor retardation (n=2) or were pregnant (n=9) (Table 2).

**Table 2:** Complications and underlying health conditions among the hospitalized measles patients (n=284) according to age groups.

	**<1 y**	**1-4 y**	**5-9 y**	**10-14 y**	**15-19 y**	**20-29 y**	**>30 y**
	**n=62 (%**)	**n=75 (%**)	**n=10 (%**)	**n=9 (%**)	**n=35**	**n=41**	**n=52**
**Bronchopneumonia (n=227**)	46 (74.2%)	61 (81.3%)	9 (90.0%)	7 (77.8%)	29 (82.9%)	35 (85.4%)	40 (76.9%)
**Diarrhea (n=111**)	33 (53.2%)	32 (42.7%)	3 (30.0%)	3 (33.3%)	8 (22.9%)	12 (29.3%)	20 (38.5%)
**Cough with expectoration (n=32**)	1 (1.6%)				6 (17.1%)	12 (29.3%)	13 (25.0%)
**Otitis (n=8**)	4 (6.4%)	3 (4.0%)					1 (1.9%)
**Laringitis (n=8**)	1 (1.6%)	1 (1.3%)			1 (2.9%)	3 (7.3%)	2 (3.8%)
**Febrile convulsions (n=4**)		4 (5.3%)					
**Epistaxis (n=2**)						2 (4.9%)	
**Chronic bronchitis (n=5**)	3 (4.8%)	2 (2.7%)					
**Asthma (n=4**)	1 (1.6%)	1 (1.3%)					2 (3.8%)
**Epilepsy (n=2**)		1 (1.3%)		1 (11.1%)			
**Psychomotor retardation (n=2**)		1 (1.3%)				1 (2.4%)	
**Pregnancy (n=9**)						6 (14.6%)	3 (5.8%)

During hospitalization chest X-ray was done in 238 patients and 227 of those (95.38%) had inflammatory infiltrates (Table 3). Most of the patients with abnormal X-ray had interstitial infiltrates (n=164, 72.25%), some had alveolar infiltrates (n=10, 4.41%) and the rest had mixed infiltrates (n=53, 23.35%). Laboratory findings are summarized in Table 3.

One fatal case was registered in May 2011. The 20 year old patient had severe bronchopneumonia and a history of co-morbidity with cerebral paralysis and chronic obstructive pulmonary disease.

## Discussion

During the recent measles outbreak cases seemed to be confined to larger cities (> 50000 inhabitants) in the North and East of the country, in particular Skopje (Figure 2). While this may be due to the higher population density in the cities and to urban pockets of susceptibles, a weaker surveillance in rural areas could also explain this finding.

In Skopje, nearly 44% of all reported cases were hospitalized (251/573, 43.80%) (Figure 3), most of them with bronchopneumonia and to a lesser extend with diarrhea (Figure 4). One patient with chronic lung disease died from pneumonia. A comprehensive study investigating measles outbreaks all over Europe in 2006 and 2007 found overall hospitalization rates of 55% and 22%, respectively [15], but sometimes rates lower than 20% [6,14] or higher than 60% were reported [16,17]. This wide range in hospitalization rates across Europe depends largely on denominators (e.g. suspected versus laboratory confirmed cases) and may also be related to differences in policies, sensitivity of surveillance systems, and the affected age groups. The relatively high hospitalization rate during the outbreak in Macedonia may be due to a combination of different aspects. Obviously younger patients with a higher risk of complications and patients with more severe symptoms were more likely to seek medical care and be registered as cases. Thus, the apparent high rate of hospitalization may in part be due to a suboptimal surveillance system that failed to detect individuals with mild disease.

**Table 3:** Laboratory and radiologic parameters of the hospitalized patients (n=284 if not stated otherwise).

**Laboratory and radiologic parameters**	
Median white blood cells per litre	7.41х10^9^ (±3.42 x10^9^)
Patients with increased Leukocytes >9x10^9^/l	68 (23.9%)
Median ESR in mm/h (n=145)	27.15 (±19.09 )
Patients with increased ESR >20 mm/h (n=145)	70 (48.3%)
Median CRP in mg/l (n=84)	54.74 (±74.82 )
Patients with CRP 10-80 mg/l (n=84)	69 (82.1%)
Patients with increased CRP >80 mg/l (n=84)	15 (17.9%)
Patients with increased ALT >40 U/L (n=192)	75 (39.1%)
Patients with ALT 81-200 U/L (n=75)	23 (30.7%)
Patients with ALT 201-400 U/L (n=75)	10 (13.3%)
Patients with increased (AST) >35 U/L (n=192)	142 (74.0%)
Patients with AST 71-175 U/L (n=142)	19 (13.4%)
Patients with AST 176-350 U/L (n=142)	22 (15.5%)
Patients with increased LDH >600 U/L (n=28)	21 (75.0%)
Patients with Proteinuria	14 (4.9%)
Patients with abnormal chest-X-Ray	227 (79.9%)
Alveolar infiltrates (n=227)	10 (4.4%)
Interstitial infiltrates (n=227)	164 (72.2%)
Mixed infiltrates (n=227)	53 (23.3%)
Anti-measles IgM positive patients (n=86)	82 (95.3%)

ESR: Erythrocyte Sedimentation Rate, CRP: C- Reactive Protein, ALT: Alanine transaminase, AST: Aspartate transaminase, LDH: Lactate dehydrogenase.

Our findings support the observation that complications are more common in children below 5 [2] since 63.4% of all cases reported in Skopje in this age group were hospitalized while only 46.4% of the 5 till 19 year olds and less than one third of the 20 years and older patients were admitted at the hospital (30.11%).

The fact that most patients (196/284, 69.01%) were hospitalized between January and April 2011 was due to the increased disease incidence during these months (Figure 3), but probably also to the higher risk of complicating respiratory super-infections during winter and early spring.

Most hospitalized patients had not been vaccinated (n=263, 92.61%) and many were of non-Macedonian nationality (n=156, 54.93%) or belonged to the Roma ethnicity (n=73, 25.70%). In addition, many patients were younger than 12 months (n=62, 21.83%) and were therefore not yet vaccinated. Thus the outbreak in Skopje was largely due to susceptible individuals with Albanian nationality, the Roma community and children below 12 months of age. Babies born to susceptible mothers lack protective maternal IgG antibodies and are susceptible essentially for one year until they receive their first dose of measles vaccine. Additional vaccination opportunities for susceptible people with non-Macedonian nationality and members of the traveler communities as well as vaccination before 12 months of age in high risk settings and situations need to be considered.

The measles virus strain responsible for the outbreak in Skopje was D4-Hamburg. This strain had been introduced from the UK to Germany by the end of 2008. Between 2009 and 2011 it spread to many European countries, including Bulgaria, Greece, Serbia and Macedonia [18]. More than 25000 cases were associated with this virus variant in different European countries and the virus spread was often linked to movements of Roma people [18]. For the recent outbreak in Skopje, the index case is not known and it is not clear from where and how the virus was introduced.

## Conclusion

During the 2010/2011 measles outbreak in the Former Yugoslav Republic of Macedonia most cases occurred in a few larger cities in the North and East of the country. The apparent hospitalization rate was relatively high, probably because of overrepresentation of the more severe cases and a high attack rate in very young children. Most patients suffered from bronchopneumonia or diarrhea. The outbreak was due to urban pockets of unvaccinated susceptibles with Albanian nationality, Roma as well as children below the age of routine vaccination. To further enhance measles control in Macedonia, additional vaccination opportunities should be offered to these groups of susceptibles.
